# Effect of Nasal Obstructive Disorders on Sinonasal Symptoms in Children with Different Levels of Bronchial Asthma Control

**DOI:** 10.1155/2018/4835823

**Published:** 2018-05-08

**Authors:** T. I. Eliseeva, S. V. Krasilnikova, N. A. Geppe, S. Yu. Babaev, E. V. Tush, O. V. Khaletskaya, D. Yu. Ovsyannikov, I. I. Balabolkin, S. K. Ignatov, N. I. Kubysheva

**Affiliations:** ^1^Department of Hospital Pediatrics and Department of ENT Diseases, Nizhny Novgorod State Medical Academy, 10/1 Minin and Pozharsky Square, Nizhny Novgorod 603005, Russia; ^2^Department of Children Diseases, I.M. Sechenov First Moscow State Medical University, 8 Bld. 2 Trubetskaya St., Moscow 119991, Russia; ^3^Department of Pediatrics, Medical Institute, Peoples' Friendship University of Russia (RUDN University), 6 Miklukho-Maklaya St., Moscow 117198, Russia; ^4^Russian Academy of Sciences, Department of Pulmonology and Allergology, National Scientific and Practical Centre of Children's Health, Lomonosovsky Avenue, Moscow 119991, Russia; ^5^Department of Chemistry, Lobachevsky State University of Nizhny Novgorod, 23 Gagarin Avenue, Nizhny Novgorod 603950, Russia; ^6^Medical Informatics Research Laboratory of the Higher School of Information Technologies and Information Systems, Kazan Federal University, 18 Kremlyovskaya St., Kazan, Republic of Tatarstan 420008, Russia

## Abstract

Allergic rhinitis (AR) and allergic rhinosinusitis (ARS) are typical upper airway pathologies (UAP) in children with bronchial asthma (BA) frequently accompanied with nasal obstructive diseases (NOD). In order to establish the effect of NOD on correlations between nasal and synonasal symptoms with clinical assessments of asthma control, 82 children, 9.8 [8.9; 10.7] years old, with atopic BA were assessed using ACQ-5 for the BA control level, TNSS for nasal symptoms, and SNOT-20 for synonasal quality of life in combination with rhinovideoendoscopy for NOD. All patients had AR/ARS; in 76.3% (63/82) of children, UAP had a multimorbid character with the presence of NOD. Significant correlations were found between ACQ-5 and TNSS (*R*=0.40, *p* < 0.0001) and ACQ-5 and SNOT-20 (*R*=0.42, *p* < 0.0001). Correlations between TNSS/ACQ-5 and SNOT-20/ACQ-5 were higher in patients who do not have a combination of AR/ARS with NOD (*R*=0.67, *p*=0.0012; *R*=0.50, *p*=0.022, resp.) than in patients who have AR/ARS combined with NOD (*R*=0.30, *p*=0.015; *R*=0.26, *p*=0.04, resp.). Thus, the association of BA control level with the expression of nasal and synonasal symptoms is higher in children who do not have multimorbid UAP.

## 1. Introduction

Bronchial asthma (BA) is the most common chronic disease of the respiratory system in children [1]. According to the Global Initiative for Asthma (GINA), the goals of asthma treatment in children and adults are to minimize both the symptom burden (day-to-day symptoms, disturbed sleep, and activity limitation) and the risk of adverse asthma outcomes (exacerbations, persistent airflow limitation, and medication side effects) [1, 2]. Together, these two domains constitute asthma control.

Assessment of the asthma control level is currently carried out mainly clinically, using validated questionnaires, for example, Asthma Control Questionnaire 5 (ACQ-5) [3]. Clinical evaluation of the asthma control level can be supplemented by the measurements of spirometric parameters and by the tests for bronchial hyperreactivity and reversibility of bronchial obstruction. However, it was noted that clinical and functional parameters often do not correlate well enough with each other [4, 5]. Attempts to use biomarkers of inflammation, both organ-specific and systemic, as control criteria are also currently the subject of scientific discussion; the results of such studies have not yet been recommended by conciliation documents for practical use [4, 6].

The basis for the asthma control management is anti-inflammatory basic therapy since the most significant component of the pathogenesis of the disease is chronic inflammation localized in the respiratory tract [7] and occurs in children patients by the Th2-dependent mechanism [1, 7]. The treatment of asthma based on this approach demonstrated significant success [8]. However, modern studies demonstrate that despite the wide arsenal of pharmacological agents, the proportion of patients who do not have the proper control characteristics can reach 56% [8, 9].

One of the reasons for the insufficient level of asthma control is the negative impact of comorbid diseases, first of all, upper airway pathology, including allergic rhinitis (AR) and allergic rhinosinusitis (ARS) [10–13]. IgE-mediated inflammation of the nasal mucosa in the structures of the osteomeatal complex leads to disorders of mucociliary transport, to drainage of mucus from paranasal sinuses, and to ventilation disorders. This, in turn, can have a negative impact on the lower respiratory tract due to the formation of the rhinobronchial reflex and cytokinemia due to the influx of inflammation products localized in the upper respiratory tract [14–16]. The generality of the formation of the inflammatory process in the mucosa of the upper and lower respiratory tract in the BA has found its reflection in the concept of “One airway, one disease” [17].

It was earlier indicated by Blaiss [18] and demonstrated in our recent studies [19, 20] that AR can occur in all children with atopic asthma who have nasal symptoms. There is an opinion that the inflammatory process in the mucosa of the nasal cavity, including the process of allergic genesis, affects the mucosa of the paranasal sinuses. Recent studies have demonstrated that the inflammatory response to nasal provocation with an allergen causes a change not only in the nasal mucosa but also in the paranasal sinuses [21–23]. On the basis of clinical data only, it is difficult to differentiate AR and ARS in asthmatic patients who have nasal symptoms [24]. So, for example, we have before found that with ultrasound sinusoscopy in 74% of children with asthma and nasal symptoms, there is a thickening of the mucosa of the maxillary paranasal sinuses in comparison with the normal [25]. It was difficult to differentiate between children with thickening of the mucous membrane and children with normal membrane thickness on the basis of clinical data only. Consequently, in the following discussion, we will not focus on the differentiation of AR and ARS.

Another factor whose influence on the joint course of the inflammatory processes of the upper respiratory tract and asthma is not yet clear is the anatomical feature of the upper respiratory tract that promote nasal obstruction. Among them, anomalies of intranasal structures and hypertrophy of the pharyngeal tonsils are most abundant. Earlier, it was found in our rhinoendoscopy study [19, 26] that 60% of children with asthma and nasal symptoms had anomalies of intranasal structures combined with AR. Also, up to 80% of patients with BA of preschool age and up to 20% of children with BA of school age had hypertrophy of the pharyngeal tonsil. Some patients with asthma had a combination of anomalies of intranasal structures and hypertrophy of the pharyngeal tonsil [27]. In recent years, there have been studies that prove the negative effect of nasal obstructive disorders (NOD) on the course and the results of AR and ARS therapy [28–30].

It should be noted that, in studies on the interaction of BA and AR/ARS, obstructive upper respiratory tract disorders are often an exclusion criterion; that is, they are deliberately removed from consideration. This fact narrows down the scope of the study since the frequency of the combination of AR/ARS and NOD in children with asthma is high according to our data.

Despite the existence of an agreed position on the negative impact of the pathology of the upper respiratory tract on the course of asthma, studies on the relationship between symptoms of the upper respiratory tract, primarily AR and ARS, with a control level of BA are scarce [24]. In the study of Huang et al. [24] and Thorstensen et al. [31], a statistically significant relationship was established between the level of control of asthma and the expression of synonasal symptoms in adult patients with asthma [24, 31]. A study by Kilaikode et al. demonstrated a link between synonasal symptoms and the level of asthma control in children, but this study did not provide a description of the pathology of the upper respiratory tract, taking into account its multimorbidity [32]. In the work of Chen et al. [33], patients with anatomical deformities affecting the patency of the upper respiratory tract were excluded from the cohort for the study of nasal symptoms, rhinomanometric indices, and markers of inflammation in asthmatic children.

Thus, according to Chawes [14], “Asthma is a common comorbidity in subjects with allergic rhinitis and epidemiological surveys have suggested a close connection between upper and lower airway diseases expressed as the “united airways concept.” Nevertheless, the nature of this association is poorly understood and there is a paucity of data objectivizing this association in children.” To our knowledge, there are no studies that take into account the influence of NOD on the association of the level of control of asthma and the severity of nasal and synonasal symptoms. At the same time, the proportion of patients with a combination of allergic diseases of the upper respiratory tract and NOD is high in the pediatric population [26–29].

In connection with the foregoing studies, the purpose of this study is to determine the relationship between nasal and synonasal symptoms with clinical assessments of the asthma control level in children, taking into account the combination of AR/ARS and NOD. In particular, we will examine how the asthma control correlates with AR/ARS symptoms in patients with and without NOD.

In this study, all patients with asthma who had clinical signs of AR did not rule out the presence of ARS with different levels of clinical manifestation. AR and ARS were considered by us as close nosologies, in the form of a single sample. In addition to AR/ARS, special attention was paid to NOD, potentially contributing to the formation and maintenance of nasal obstruction—abnormalities of intranasal structures (including curvature of the nasal septum) and hypertrophic changes in the pharyngeal tonsil of significant degrees (overlapping not less than ½ of the clearance of the khoan) and their combination.

## 2. Methods

### 2.1. Patients

The study was conducted according to the Helsinki Declaration adopted in June 1964 (Helsinki, Finland) and revised in October 2000 (Edinburgh, Scotland) and approved by the Ethics Committee of the Nizhny Novgorod State Medical University, protocol number 13 of 10.10.16. Informed consents were obtained from the patients between 15 and 17 years old and from the parents of patients under the age of 15 years, according to the Federal Law “Fundamentals of the Legislation of the Russian Federation on the Protection of Health of Citizens” of July 22, 1993, number 5487-1.

A total of 82 children and adolescents aged from 5 to 17 years were examined (the average age 9.8 [8.9; 10.7] years; boys 67.0% (55/82)) who were on treatment at the Children's Clinical Hospital No. 1 in Nizhny Novgorod for asthma and who had nasal or synonymous complaints (symptoms). All children had a symptomatic complex that was characteristic of BA and AR. The family anamnesis associated with atopy (asthma, AR, conjunctivitis, atopic dermatitis, and urticaria) was evaluated in all children. The etiology of allergy was established on the basis of the following studies: (1) assessment of the impact on the occurrence of symptoms of rhinitis and/or bronchial asthma of respiratory allergens (house dust, house dust mites, library dust, animal allergens, and plant pollen); (2) positive skin prick tests with the main aeroallergens characteristic for the Volga–Vyatka region of the Russian Federation and/or the presence of high titers of class E–specific immunoglobulins for at least one of these allergens. The survey was carried out in the autumn–winter period—in October–December 2017, which minimized the influence of pollen allergens.

Criteria for inclusion were as follows: a diagnosis of BA, delivered by existing international and national conciliation documents, and the presence of nasal or synonymous complaints and symptoms in patients.

Exclusion criteria include the presence of acute infectious diseases and fever, diabetes, autoimmune disorders, primary immunodeficiencies, and oncology.

Treatment of asthma and related diseases of the upper respiratory tract was conducted by the existing consensus documents with current therapeutic strategies [1, 8, 34].

### 2.2. Objective and Subjective Measurements

Verification of the diagnosis of BA was carried out by the available recommendations [1]. All patients underwent a general clinical examination with a quantitative assessment of the level of control of asthma using the ACQ-5 test. This test includes 5 questions reflecting the key clinical parameters of asthma control, each of which is evaluated on a 6-point scale from 0 points (no symptom) to 5 points (greatest symptom severity); the results are summarized and divided by the number of questions, and the evaluation was conducted over a period of one previous week. The test values from 0 to 0.75 testify to the achieved control of asthma, from 0.75 to about 1.5—about the partial level of disease control, from 1.5 points and above—about the absence of disease control. In this study, patients were identified with the achieved control of asthma (ACQ-5 less than 0.75 points). Patients with partial control and lack of control of asthma were combined into single group (ACQ-5 more than 0.75 points). The results of the tests were compared with the clinical assessment of the control by an experienced allergist by the control criteria set out in the GINA.

Spirographic studies were performed using the MasterScreen Pneumo spirometer (Jaeger, Germany) by existing international guidelines [35]. The forced vital capacity (FVC), forced expiratory volume per second (FEV1), and the ratio FEV1/FVC were evaluated; the data were recorded both in absolute values of the indices and in relative units (in comparison with the relevant values determined, taking into account age, sex, growth children, and their ethnicity) [35].

All BA patients were examined by an otorhinolaryngologist. They carried out a routine otorhinolaryngological examination in conjunction with the testing of TNSS and SNOT-20.

Total Nasal Symptom Score (TNSS), a scale for assessing symptoms of rhinitis, includes four questions (severity of rhinorrhea, sneezing, itching, and nasal obstruction). Each symptom is assessed on a 4-point scale from 0 points (no symptom) to 4 points (most symptom severity) with the next determination of the total score [36].

To assess the quality of life of patients with rhinosinusitis, Sino-Nasal Outcome Test-20 (SNOT-20) is used. This test includes 20 questions, each of which is evaluated on a 6-point scale from 0 to 5 points. The questions are combined into blocks evaluating the most important syndromal characteristics that determine the quality of life of patients with rhinosinusitis: rhinologic, ear/facial, sleep function, and psychological function [37].

Diagnosis of allergic rhinitis and allergic rhinosinusitis was given in accordance with the available international recommendations. The involvement of sinuses in the pathological process was noted using the criteria of the European Position Paper on Rhinosinusitis and Nasal Polyps (EPOS)–2012 [38]. In assessing the pathology of the upper respiratory tract, the diagnosis was verified using the International Classification of Diseases X revision, the recommendations of the Allergic Rhinitis and its Impact on Asthma (ARIA) 2008 update, the International Consensus on the Diagnosis and Treatment of Rhinitis, the European Position Paper on Rhinosinusitis and Nasal Polyps, and the classification of the pathology of the lymphoepithelial ring of the pharynx [38–41].

Hypertrophic changes in the mucous membrane of the nasal cavity, hypertrophic changes in the pharyngeal tonsil, and anomalies of the intranasal structures were assessed by means of rhino and video endoscopy using endoscopic techniques and endoscopic photography. Rigid rhinoscopes from the company Karl Storz (Germany) with a viewing angle of 0° and 30°and diameter of 2.7 and 4.0 mm were used; video laryngoscopy was performed by a 90° laryngoscope (Atmos, Germany) and a flexible nasopharyngoscope 3.2 mm; video fixation—a video camera (Atmos, Germany). Rhinosovideoscopic examination was performed after instillation of a 2% solution of lidocaine on the nasal mucosa and application anesthesia using 0.1% solution of epinephrine hydrochloride and 10% lidocaine.

According to the testimony, individual patients underwent X-ray examinations by international recommendations on sinusitis [38].

### 2.3. Statistical Analysis

The statistical analysis was carried out using the Statgraphics Centurion software package, v.9. The data are presented in the form of Me [Q1; Q2], where Me is the median and [Q1; Q2] is the 95% confidence interval. Continuous variables were compared between groups using the statistical criteria based on Student's *t*-test and Kolmogorov–Smirnov tests. Correlations between the two sets were estimated using the Spearman correlation coefficient. Statistically significant values were considered at *p* < 0.05.

## 3. Results

### 3.1. Clinical Characteristics of Patients

Demographic, spirometric measurements, and results of assessing the level of control of asthma and the characteristics of nasal and rhinonasal symptoms of the patients examined are shown in Tables 1 and 2.

In all patients, allergic rhinitis (AR) was verified, both persistent 72/82 (87.8%) and intermittent 10/82 (12.2%). This is consistent with both our before published data and data provided by Blaiss [18, 19, 27].

The severity of AR flow was as follows: lung course AR was diagnosed in 7/82 (8.5%) patients, moderate-to-severe AR was diagnosed in 70/82 (85.4%) children, and severe AP was in 5/82 (6.1%). Also, 71/82 (86.6%) children with asthma have a multimorbid pathology of the upper respiratory tract. Disturbances in the architectonics of the nose were found in 40/82 (48.8%) patients, and the pathology of the nasopharyngeal tonsil was in 48/82 (58.8%) children. The combination of AR with chronic nonspecific rhinitis was diagnosed in 5/82 patients (6.1%). Patients of this group were characterized by persistence of pathogenic and opportunistic flora on the nasal mucosa. Hypertrophic changes in the nasal mucosa were present in 4/82 (4.8%) of the examined children with asthma. The pathology of palatine tonsils was detected in 19/82 (23.2%) patients.

Patients with asthma and allergic rhinitis without anomalies of intranasal structures and hypertrophy of the pharyngeal lymphoid ring accounted for group 1 (19 patients). The remaining 63 patients, in addition to the symptoms of AR, had nasal obstructive disorders: a violation of the architectonics of the nasal cavity due to anomalies of the bone-cartilaginous skeleton of the nose—40 children; adenoids of significant degrees—48 children; and a combination of abnormalities of intranasal structures and hypertrophy of the pharyngeal tonsil—21 children [29, 42]. These 63 patients were grouped into group 2.

Thus, it was demonstrated that, for children and adolescents with atopic asthma and nasal symptoms, the symptoms of AR predominate in combination with other variants of the pathology of the upper respiratory tract. This, in our opinion, they must be taken into account when managing patients with atopic asthma.

### 3.2. Correlation between Tests Characterizing the State of the Upper Respiratory Tract and Tests Reflecting the Asthma Control Level

Given the combination of atopic asthma with VAD pathology in the examined patients, it can be assumed that the level of control of asthma can be related to the severity of symptoms of rhinitis and/or rhinosinusitis, a quantitative assessment of which is possible with the use of appropriate tests—TNSS and SNOT-20. We studied the correlation between the results of tests that reflect the asthma control level, ACQ-5, on the one hand, and TNSS and SNOT-20 tests, which characterize nasal and synonasal symptoms, on the other hand. These associations were studied by taking into account the division of patients into two groups, distinguished on the basis of the absence or presence of NOD.

**Figure 1:**
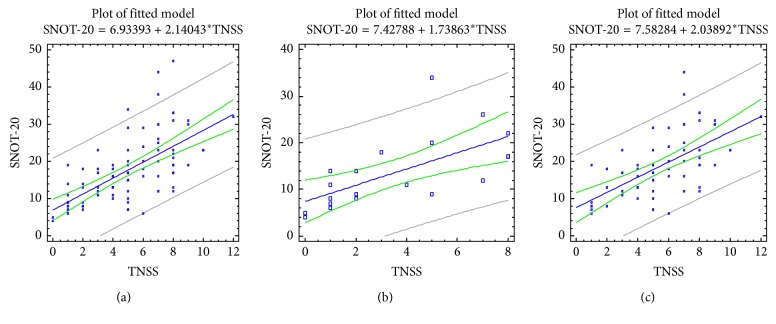
Correlations between TNSS and SNOT-20 scores in patients with asthma, taking into account the comorbid pathology of the upper airways. (a) All patients; (b) children with bronchial asthma and AR/ARS; (c) children with bronchial asthma, AR/ARS, and NOD.

A significant correlation was found between the tests evaluating the severity of nasal and synonasal symptoms (TNSS and SNOT-20 tests); the correlation coefficient between the TNSS and SNOT-20 tests was *R*=0.66  for  *p* < 0.0001 (Table 3 and Figure 1). Correlation levels between TNSS and SNOT-20 tests were comparable in patients of both groups, both with the absence (*R*=0.65, *p*=0.002) and with the presence of NOD (*R*=0.62, *p* < 0.0001).

**Figure 2:**
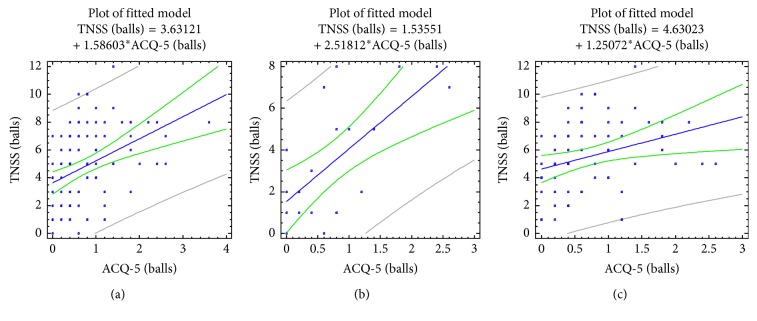
Correlations between the BA control level (ACQ-5 scores) and nasal symptoms (TNSS) in patients with asthma, taking into account the comorbid pathology of the upper airways. (a) All patients; (b) children with bronchial asthma and AR/ARS; (c) children with bronchial asthma, AR/ARS, and NOD.

**Figure 3:**
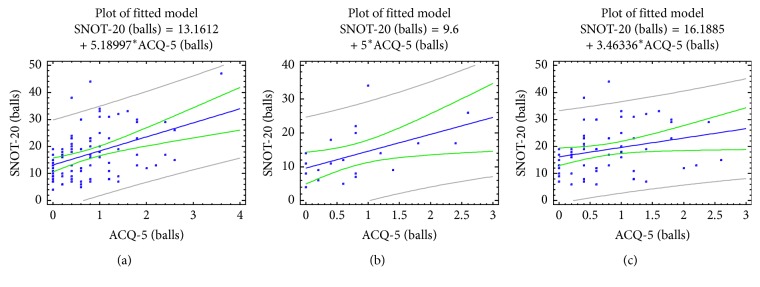
Correlations between the BA control level (ACQ-5 scores) and synonasal symptoms (SNOT-20, scores) in patients with BA, taking into account the comorbid pathology of the upper airways. (a) All patients; (b) children with bronchial asthma and AR/ARS; (c) children with bronchial asthma, AR/ARS, and NOD.

The relationship between the results of a clinical assessment of the level of control of asthma using the ACQ-5 test with nasal (TNSS) and synonasal (SNOT-20) symptoms is also statistically significant (Table 3 and Figures 2 and 3). Correlation coefficients were *R*=0.40 with *p*=0.0001 for TNSS and *R*=0.42 with *p*=0.0001 for SNOT-20. This indicates an association between the level of control of asthma and the expression of nasal and synonasal symptoms in this contingent of patients. The association of nasal symptoms (TNSS) and synonasal quality of life (SNOT-20) with the level of control of asthma (ACQ-5) is higher in patients of the first group who do not have a combination of AR with NOD than in patients in group 2, having a combination of asthma with both AR and NOD.

Thus, the results obtained show that the presence of pathology from the group of nasal obstructive disorders in children with asthma and AR has a significant effect on the relationship of symptoms of asthma with nasal and synonasal symptoms.

### 3.3. Detailing the Relationship between TNSS and SNOT-20 with the Level of Control of Asthma

Considering that the TNSS and SNOT-20 scales are represented by a set of questions reflecting the different clinical aspects of rhinitis, rhinosinusitis, and related quality of life, a detailed study was carried out on the relationship of individual components of these questionnaires to the level of control of asthma assessed using the ACQ-5 scale.

When considering the nature of the relationship between the results of the ASQ-5 test and the severity of each of the symptoms, which together make up the TNSS test, it was established that the greatest correlation of the control level of BA occurs with rhinorrhea (*R*=0.38, *p*=0.0002) and sneezing (*R*=0.37, *p*=0.0003), somewhat smaller with nasal obstruction (*R*=0.25, *p*=0.019), but there was no clear correlation between the level of control of asthma with nasal itching (*R*=0.19, *p*=0.07) (Table 4). However, the relationship between the expression of individual symptoms of AR (with the exception of pruritus) and the level of control of BA is more typical for the patient of the first group without anomalies of intranasal structures and/or hypertrophy of the pharyngeal tonsil. Moreover, this relationship is significantly weaker or virtually absent in patients of the second group who have a combination of AR and NOD.

When considering SNOT-20, it is customary to single out four blocks of questions: (1) rhinological block reflecting the expression of complaints associated with rhinitis; (2) ear/facial block reflecting the severity of symptoms indicative of involvement in the middle ear process and/or the presence of facial pain associated with paranasal sinuses; (3) sleep function block reflecting the influence of synonasal symptoms on sleep quality; and (4) a complex of psychoasthenic or vegetative symptoms (psychological function).

**Figure 4:**
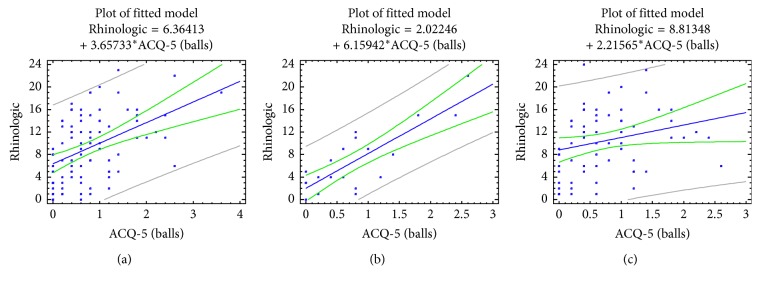
Correlations between BA control level (ACQ-5 scores) and the rhinological block of SNOT-20 symptoms in patients with asthma, taking into account the comorbid pathology of the upper respiratory tract. (a) All patients; (b) children with bronchial asthma and AR/ARS; (c) children with bronchial asthma, AR/ARS, and NOD.

Investigation of the relationship between the level of control of asthma and the results of SNOT-20 for each of these four blocks of questions is presented in Table 5. It is clear that the most clear link with the level of control of asthma is noted in the block of rhinological symptoms (*R*=0.46, *p* < 0.0001) in SNOT-20. Moreover, this association is significantly stronger among patients with asthma and AR who do not have nasal obstructive disorders (*R*=0.82, *p* < 0.0001), compared with patients of the second group (*R*=0.25, *p*=0.049), Figure 4.

The results obtained for the block of ear/facial symptoms in general do not show a connection with the level of control of asthma, but in patients who have a combination of asthma with AR and with NOD, there is a tendency to have a connection between the level of control of BA and this symptoms block (*R*=0.21, *p*=0.096). It can be assumed that the increase in allergic inflammation in the respiratory tract in children with no control of asthma (in the period of exacerbation of asthma) in the presence of initial anatomical features in the form of anomalies of intranasal structures and/or hypertrophic pharyngeal tonsil can provoke or strengthen symptoms from the middle ear and/or lead to the emergence of facial pain at characteristic points.

Symptoms that indicate a decrease in sleep quality have a statistically significant relationship with the level of control of asthma (*R*=0.32, *p*=0.0013), which, overall, is expected. No psychological symptoms were associated with the level of control of asthma (*R*=−0.02, *p*=0.82).

It was noted above that rhinological symptoms show a clear relationship with the asthma control level. From the list of symptoms composing this block, the greatest interrelation with ACQ-5 is traced with a cough (*R*=0.55, *p* < 0.0001), which can be considered in the context of cough as a universal symptom both in relation to asthma and in relation to AR and ARS (Table 6). It is obvious that such a symptom as a cough can have a combined genesis and be a reflection of both the synonasal symptoms and the BA itself. Differential diagnosis of the genesis of cough in these patients is very complicated. The distinct correlation of ACQ-5 is also established with symptoms such as nasal discharge, the need to freshen up. In this case, the revealed associative relationships dominate in children of the first group who do not have NOD.

The association of ear/facial symptoms as a whole with the level of control of asthma in the sample of patients was not revealed (*R*=0.1, *p*=0.32). However, for patients in the second group with a combination of BA + AR + NOD, association with the level of AD control for symptoms such as dizziness and ear pain was significant (Table 6).

It should be noted that the correlation between the level of control of asthma and night awakenings was found in the children of the first group, which can also be considered as a component of an insufficient level of control of asthma (Table 6). This association in the group of children with BA + AR + NOD also occurs, but much less pronounced. For other symptoms that make up this block of the SNOT-20 questionnaire, the relationship with the level of control of asthma is not established.

In this study, there was no correlation between the level of control of BA and the block of psychovegetative symptoms and its components (Table 6).

Obviously, with regard to assessing the relationship of SNOT-20 symptoms with the level of control of asthma, nasal symptoms (*p* < 0.0001) are most important and, to a lesser degree, some of the symptoms associated with sleep quality (*p*=0.0013). At the same time, symptoms that reflect the psycho-vegetative characteristics of the patient (symptoms of shedding) seem to be neglected since the relationship between the level of control of asthma and these symptoms is not established.

With the exclusion of the symptoms characterizing the psychological state of the patient from the SNOT-20 complex, the relationship of the combination of symptoms characterizing the nasal symptoms, the quality of night sleep, and ear/facial symptoms, with the level of control of the BA as a whole does not change and even appreciably increases in each of the groups to *R*=0.73  at  *p*=0.0005 in the first group and to *R*=0.37  at  *p*=0.0028 in the second group of patients.

Thus, with regard to the synonasal symptoms in patients with atopic asthma, the exclusion of SNOT-20 symptoms that do not show an association with the level of control of asthma allows this questionnaire to be reduced to 14 most relevant questions for asthma. The correlation level of ACQ-5 is not changed in the sample, and even increases in each of the groups under consideration.

### 3.4. Severity of Symptoms of Upper Respiratory Tract Pathology in Patients with Different Levels of Control of Asthma

The established correlations between the values of the ACQ-5 test and the tests of TNSS and SNOT-20 make it possible to compare the severity of nasal and synonasal symptoms from the upper respiratory tract in children with different levels of BA control (Table 7). It has been established that as the level of BA control decreases, there is a progressive increase in the symptoms of AR and ARS in these patients, which is manifested by an increase in the values of the TNSS and SNOT-20 tests in scores. Differences are statistically significant: *p*=0.009 and *p*=0.002, respectively. This is consistent with the views on the negative effects of rhinitis and rhinosinusitis on achieving control of asthma [43].

It should also be noted that, among the children with BA control lack, the expression of nasal and synonasal symptoms in the groups 1 (BA + AR) and 2 (BA + AR + NOD) is comparable. At the same time, in children with achieved BA control, the severity of nasal and synonasal symptoms in the group 2 is higher than in the group 1. This indicates that in children with multimorbid pathology of the upper respiratory tract, proper reduction of nasal and synonasal symptoms is not observed, even if the control of asthma is reached.

## 4. Discussion

In the present study, the comparison of the asthma control level estimated using the ACQ-5 questionnaire, and the expression of nasal symptoms (TNSS test) and the synonasal quality of life (SNOT-20 test) in patients of the two groups with the absence and presence of NOD, including anomalies of intranasal structures, hypertrophy of the pharyngeal tonsil, or a combination of these pathological conditions, were assessed.

It has been established that the level of control of asthma has a significant direct correlation with the severity of synonasal symptoms in patients of childhood. As the level of BA control decreases, there is an increase in nasal and synonasal symptoms. These results are consistent with the concept of “One airway, one disease” and testify to a potential commonality of involvement in the pathological process in the BA of the upper and lower respiratory tract [17]. The new data obtained are in good agreement with the studies of Huang et al. [24], Thorstensen et al. [31], and Kilaikode et al. [32], which stress the importance of upper respiratory pathology in the management of patients with asthma. Thus, our study is a confirmation of the concept of “United Airways,” which indicates that for patients with inflammation of the lower respiratory tract, parallel inflammation in the upper respiratory tract is characteristic.

In addition, we found that the relationship between the BA control level with nasal and synonasal symptoms is higher in patients who do not have multimorbid pathology from the upper respiratory tract. In those children with asthma who have a combination of AR/ARS with NOD, this relationship is less pronounced. These patterns are demonstrated for the first time.

In the current study, the children were divided by the BA control level into patients with achieved control (ACQ-5 less than 0.75 points) and with unreached control (ACQ-5 more than 0.75 points). Both in the joint group of patients, without taking into account the multimorbidity of upper respiratory tract pathologies, and in separate groups with and without NOD, the expression of nasal and synonasal symptoms is higher in children with unreached control compared with children who have complete control of asthma. However, in the group of the achieved control, the severity of nasal and synonasal symptoms in children with multimorbid pathology of the upper respiratory tract is higher than in children who do not have nasal obstructive disorders.

This indicates that the multimorbid pathology of the upper respiratory tract in children with BA may disrupt the cyclicity of nasal and synonasal symptoms depending on the level of BA control, inducing resistance to the therapy of symptoms of the upper respiratory tract, even under the conditions of the control of asthma.

When considering the correlation between the BA control level with the components of the TNSS and SNOT-20 tests, it was found that the most pronounced correlation of the ACQ-5 test scores was obtained with rhinorrhea, sneezing, nasal obstruction, but not itching.

Among the blocks of questions of SNOT-20, the largest association of the results of the ACQ-5 test is found with the rhinological block and, to a lesser extent, with the quality of sleep. Ear/facial syndrome and psychological syndrome did not demonstrate a clear association with ACQ-5 scores.

The results of the evaluation of the SNOT-20 test in children with different levels of BA control indicate a correlation of synonasal symptoms and the BA control level. At present, the complexity of differentiation between AR and ARS is obvious with the use of only clinical manifestations (both subjective and objective) in patients with asthma who have nasal symptoms [24]. Thus, for example, in our studies, it was found that 74% of children with ultrasound examination of the thickness of the mucous membrane of the maxillary sinuses noted its excess compared with a healthy control [25]. In a study by Huang et al., about half (52%) of patients with bronchial asthma and nasal symptoms had chronic rhinosinusitis [24]. Thus, in our opinion, the development of algorithms is required to objectify the involvement of paranasal sinus allergic inflammation in patients with asthma, including such diagnostic methods as screening ultrasound evaluation of symptoms and endoscopy.

In our study, it was also found that the correlation degree between the expression of nasal and synonasal symptoms and the level of control of asthma is different in children who do not have and have nasal obstructive disorders. The share of the latter among the children of childhood with asthma and nasal symptoms prevails (in this study, 76.8% (63/82)). This emphasizes the desirability of a detailed study of the upper respiratory tract in children with asthma with the involvement of modern technologies and the study of the influence of NOD on the objective parameters of the level of asthma control and nasal patency.

The retention of nasal and synonasal symptoms, identified in the present study, in children with nasal obstructive disorders and with the achieved BA control should also be pointed out. This requires additional studying since the retention of nasal symptoms can potentially affect the quality of control achieved. Obviously, dynamic monitoring of the subsequent course of asthma in this group of patients is necessary in comparison with children who do not have nasal obstructive disorders.

Our study included a heterogeneous group of patients with asthma who have various, including multimorbid, conditions of the upper respiratory tract. Nevertheless, the heterogeneity of the population of our sample of patients reflects the reality of daily clinical practice. It is important to note that we have demonstrated a higher expression of nasal and synonasal symptoms in children with multimorbid conditions of the upper respiratory tract and children with only BA and AR (ARS) at the achieved control level of asthma. At the same time, nasal and sinonasal symptoms have a more pronounced correlation with subjective assessments of asthma control also among children with asthma who do not have multimorbid pathology from the upper respiratory tract. This can be a consequence of a “disruption in cyclicity” in the dynamics of nasal symptoms as the quality of asthma control is improved in children with multimorbid diseases of the upper respiratory tract and also be an evidence for the preservation of nasal and synonasal symptoms even in children with achieved control of asthma.

## 5. Conclusion

The BA control level has a clear correlation with the expression of nasal and synonasal symptoms in children. This emphasizes the need for a unified approach to consideration of allergic inflammation of the upper and lower respiratory tract in BA children and demonstrates the importance of their simultaneous therapeutic correction. At the same time, the multimorbidity of the upper respiratory tract has a significant effect on this relationship. In the conditions of achieved BA control in patients with multimorbid pathology of the upper respiratory tract, a higher level of expression of nasal and synonasal symptoms has been found. This requires close attention to the multimorbidity of nasal pathology in children with asthma and a careful studying for the effect of insufficient reduction of nasal and synonasal symptoms in children with multimorbid upper respiratory tract diseases on the control level and the course of BA in these patients.

## Figures and Tables

**Table 1:** Characteristics of patients.

Characteristics	Median and 95% confidence interval
Age (years)	10.8 [5.0; 16.0]
Boys (%)	67.0% (55/82)
BMI boys (kg/m^2^)	19.5 [18.5; 20.5]
BMI girls (kg/m^2^)	19.4 [16.8; 21.9]
ACQ-5 (points)	0.9 [0.0; 2.6]
TNSS (points)	4.8 [1.0; 9.0]
SNOT-20 (points)	17.5 [5.0; 34.0]
FEV1 (%)	90.7 [74.0; 112.0]

**Table 2:** Structure of the pathology of the upper respiratory tract in children with atopic bronchial asthma.

Diagnosis	Number of observations
All diagnoses	82
Allergic rhinitis	82 (100%)
Chronic nonspecific infectious rhinitis	5 (6.1%)
Hypertrophic rhinitis	4 (4.8%)
Anomalies of intranasal structures	40 (48.8%)
Hypertrophy of the nasopharyngeal tonsil	48 (58.5%)
Pathology of palatine tonsils	19 (23.2%)
Chronic pharyngitis	1 (1.2%)
Pathology of the larynx	1 (1.2%)

**Table 3:** Correlation relationship between the results of TNSS, SNOT-20, and ACQ-5 tests in patients with asthma, taking into account the comorbid pathology of the upper respiratory tract.

Correlation	All patients	Group 1 (BA + AR), *N* = 19	Group (BA + AR + NOD), *N* = 63
TNSS/SNOT-20	0.66 (<0.0001)	0.65 (0.002)	0.62 (<0.0001)
ACQ-5/TNSS	0.40 (0.0001)	0.67 (0.0012)	0.30 (0.015)
ACQ-5/SNOT-20	0.42 (<0.0001)	0.50 (0.022)	0.26 (0.04)

The values of the determination coefficient *R* and the significance level *p* (in parentheses) are given; TNSS: Total Nasal Symptom Score (points); SNOT-20: Sino-Nasal Outcome Test 20 (points); ACQ-5: Asthma Control Questionnaire 5 (points); BA: bronchial asthma; AR: allergic rhinitis; NOD: nasal obstructive disorders.

**Table 4:** Interrelation of the level of control of asthma (ACQ-5 scores) and symptoms included in TNSS (points).

Nasal symptoms	All patients	Group 1 (BA + AR), *N* = 19	Group 2 (BA + AR + NOD), *N* = 63
Rhinorrhea	0.38 (0.0002)	0.74 (0.0002)	0.25 (0.04)
Itching	0.19 (0.07)	0.16 (0.48)	0.16 (0.20)
Sneezing	0.37 (0.0003)	0.51 (0.02)	0.30 (0.019)
Nasal congestion	0.25 (0.019)	0.54 (0.014)	0.13 (0.32)
Total nasal symptoms	0.40 (0.0001)	0.67 (0.0012)	0.30 (0.015)

The values of the determination coefficient *R* and the significance level *p* (in parentheses) are given.

**Table 5:** The relationship between the level of control of asthma (ACQ-5 scores) and the blocks of symptoms included in SNOT-20 (points).

The blocks of symptoms included in SNOT-20	All patients	Group 1 (BA + AR) *N* = 19	Group 2 (BA + AR + NOD) *N* = 63
Nasal symptoms	0.46 (<0.0001)	0.82 (<0.0001)	0.25 (0.049)
Ear/facial symptoms	0.10 (0.32)	0.06 (0.80)	0.21 (0.096)
Sleep function	0.32 (0.0013)	0.40 (0.10)	0.14 (0.26)
Psychological function	−0.02 (0.82)	−0.35 (0.12)	0.03 (0.80)
All symptoms except psychological function	0.43 (0.0001)	0.73 (0.0005)	0.37 (0.0028)
All symptoms	0.42 (<0.0001)	0.50 (0.022)	0.26 (0.04)

The values of the determination coefficient *R* and the significance level *p* (in parentheses) are given.

**Table 6:** The relationship between the level of control of asthma (ACQ-5 scores) and individual symptoms included in SNOT-20 (points).

The blocks of symptoms included in SNOT-20	All patients	Group 1 (BA + AR), *N* = 19	Group 2 (BA + AR + NOD), *N* = 63
*Nasal symptoms*			
Need to blow nose	0.35 (0.0008)	0.76 (0.001)	0.16 (0.21)
Sneezing	0.28 (0.0075)	0.49 (0.023)	0.19 (0.13)
Runny nose	0.36 (0.0005)	0.59 (0.006)	0.17 (0.17)
Cough	0.55 (<0.0001)	0.80 (<0.0001)	0.37 (0.024)
Postnasal discharge	0.12 (0.24)	0.54 (0.031)	–0.04 (0.75)
Thick nasal discharge	0.37 (0.0003)	0.76 (0.0001)	0.16 (0.20)
*Ear/facial symptoms*			
Ear fullness	−0.07 (0.56)	No symptoms	–0.06 (0.61)
Dizziness	0.18 (0.09)	0.06 (0.80)	0.33 (0.0076)
Ear pain	0.17 (0.10)	0.06 (0.80)	0.30 (0.014)
Facial pain/pressure	0.03 (0.77)	0.06 (0.80)	0.03 (0.082)
Ear involvement/facial symptoms	0.10 (0.32)	0.06 (0.80)	0.21 (0.096)
*Symptoms related to sleep quality*			
Difficulty falling asleep	0.18 (0.09)	–0.05 (0.84)	0.08 (0.51)
Wake up at night	0.45 (<0.0001)	0.53 (0.016)	0.25 (0.045)
Lack of a good night's sleep	0.16 (0.14)	–0.26 (0.27)	0.09 (0.49)
Wake up tired	0.18 (0.09)	–0.027 (0.92)	–0.03 (0.84)
*Psychological (asthenic) symptoms*			
Fatigue	0.08 (0.44)	−0.21 (0.37)	0.12 (0.33)
Reduced productivity	−0.01 (0.93)	−0.23 (0.32)	0.03 (0.83)
Reduced concentration	−0.04 (0.96)	−0.02 (0.91)	−0.11 (0.40)
Frustrated/restless/irritable	0.11 (0.33)	−0.37 (0.1)	0.01 (0.96)
Sad	0.03 (0.80)	−0.128 (0.60)	−0.13 (0.59)
Embarrassed	−0.08 (0.44)	−0.24 (0.31)	−0.016 (0.90)

The values of the determination coefficient *R* and the significance level *p* (in parentheses) are given.

**Table 7:** The values of the TNSS scores, SNOT-20 scores, and the shortened version of SNOT-20 in patients with different levels of BA control, quantified using the ACQ-5 test.

Patient groups	Complete control of asthma (ACQ-5 < 0.75)	Poor control of asthma (ACQ-5 > 0.75)	Statistics of intergroup differences (*t*: Student's *t*-test; K-ST: Kolmogorov–Smirnov test)
*FEV1 (%)*			
All patients	92.45 [88.82; 96.06]	84.73 [79.25; 90.21]	*T* = 2.53, *p*=0.015
	(*n*=46)	(*n*=37)	K-ST = 1.42, *p*=0.036
*TNSS test results, scores*			
All patients	4.52 [3.74; 55.31]	6.03 [5.12; 6.93]	*t* = −2.55, *p*=0.013
	(*n*=46)	(*n*=37)	K-ST = 1.85, *p*=0.002
Group 1	2.22 [0.51; 3.93]	5.00 [2.98; 7.02]	*t* = −2.36, *p*=0.03
	(*n*=9)	(*n*=10)	K-ST = 1.28, *p*=0.07
Group 2	5.08 [4.27; 5.90]	6.41 [5.37; 7.45]	*t* = −2.08, *p*=0.042
	(*n*=37)	(*n*=27)	K-ST = 1.47, *p*=0.026
Differences (group 1 − group 2)	*t* = −3.19, *p*=0.0026	*t* = −1.41, *p*=0.17	
	K-ST = 1.66, *p*=0.008	K-ST = 1.02, *p*=0.25	
*SNOT-20 test results, scores*			
All patients	15.27 [13.00; 17.54]	20.84 [17.84; 23.84]	*t* = −3.04, *p*=0.003
	(*n*=46)	(*n*=37)	K-ST = 1.5, *p*=0.02
Group 1	10.22 [6.87; 13.57]	17.40 [11.29; 23.51]	*t* = −2.26, *p*=0.037
	(*n*=9)	(*n*=10)	K-ST = 1.28, *p*=0.07
Group 2	16.30 [13.72; 18.89]	22.11 [18.56; 25.66]	*t* = −2.77, *p*=0.007
	(*n*=37)	(*n*=27)	K-ST = 1.49, *p*=0.023
Differences (group 1 − group 2)	*t* = −2.28, *p*=0.027	*t* = −1.45, *p*=0.16	
	K-ST = 1.34, *p*=0.054	K-ST = 0.86, *p*=0.46	
*SNOT-20 test results without psychological block, scores*			
All patients	10.87 [8.94; 12.80]	15.95 [13.53; 18.36]	*t* = −3.36, *p*=0.001
	(*n*=46)	(*n*=37)	K-ST = 1.71, *p*=0.006
Group 1	5.11 [2.89; 7.33]	13.20 [7.83; 18.57]	*t* = −3.03, *p*=0.008
	(*n*=9)	(*n*=10)	K-ST = 1.52, *p*=0.019
Group 2	12.31 [10.18; 14.43]	17.00 [14.24; 19.76]	*t* = −2.8, *p*=0.006
	(*n*=37)	(*n*=27)	K-ST = 1.49, *p*=0.02
Differences group 1 − group 2	*t* = −3.33, *p*=0.0018	*t* = −1.45, *p*=0.15	
	K-ST = 1.64, *p*=0.009	K-ST = 1.01, *p*=0.26	

FEV1: the volume of the forced end in 1 second (% of the required values); *n,* the number of patients in the group.

## Data Availability

The data used to support the findings of this study are available from the corresponding author upon request.
